# Citizen science: An alternative way for water monitoring in Hong Kong

**DOI:** 10.1371/journal.pone.0238349

**Published:** 2020-09-08

**Authors:** Simon Yat-Fan Ho, Steven Jingliang Xu, Fred Wang-Fat Lee

**Affiliations:** Department of Science, School of Science and Technology, The Open University of Hong Kong, Hong Kong SAR, China; University of Bucharest, ROMANIA

## Abstract

Nowadays, citizen science has become increasingly popular, especially in Western countries. In Hong Kong, citizen science projects are mostly used for public education, while utilizing citizen scientists in published scientific research is very rare. On the other hand, with the increasing threats to global water security, Hong Kong requires new adaptation and strategy in facing the impairment of local freshwater systems. However, unfortunately, the number of full-scale urban river research appears to be declining. In this regard, citizen science can offer an alternative option as one of the new integrated water management strategies in Hong Kong. In this study, the water quality of seven rivers and streams in Hong Kong was studied monthly for two years by a group of citizen scientists. The main goal is to examine the reliability of data collected by citizen scientists by comparing it with the official data from the Environmental Protection Department of Hong Kong (EPD). Results show that the water temperature and conductivity data acquired by the citizen scientists were highly comparable to the official data. Also, moderate to strong correlations in water pH, turbidity, and dissolved oxygen levels were found between citizen scientists and official data. Since the citizen science data remained as high as 70% of relevance to the official data, we believe that this may serve as a supplement to the lacking official or professional water quality monitoring data in Hong Kong. Even though the use of volunteer data in water quality monitoring unavoidably exists with errors and bias, this study demonstrates a successful outcome of utilizing citizen science programme in urban river monitoring in Hong Kong.

## Introduction

The involvement of citizen scientists in various water-related research projects is popular among Western countries. The participation of citizen scientists can enhance the society linkage within the communities and seek profound bonding between human and their living environment. The USA, for example, has a long history of citizen volunteer-oriented programmes in Lake George, New York. A citizen turbidity monitoring programme taking place since 1986 was initially meant for targeting better sampling coverage of the whole lake due to limited state budget, however, it has been continuously raising public awareness of the water condition and the causes of lake eutrophication for more than 23 years [1]. The advantage of using citizen scientists is not only to provide additional manpower to large-scale researches but also serves well as the educational purpose and promotes unique practical science experience to young generations. In Sweden, thousands of high school students and teachers have been involved in a citizen science project together with the researchers. They examined the influence of greenhouse gases from inland waters on global warming. The successful outcome of this project was proved to be beneficial to both students and the researchers [2]. Abbott et al. [3] successfully used 18 years of riverine nutrient data which were collected by secondary school students and community volunteers to assess how improvements in land management affect the interannual trends and seasonality of river nutrient concentrations in western France. In the U.K., McGoff et al. [4] suggested that even an ad-hoc citizen science programme could provide information that broadly characterises the freshwater regime in urban areas. In the USA, Edwards et al. [5] demonstrated that citizen scientists can generate meaningful data in a collaborative programme aimed at monitoring the impact of river restoration construction disturbances on large invertebrate communities.

The participation of citizen scientists in water quality monitoring can complement traditional monitoring methods and has other potential advantages such as lowering monitoring costs, significantly increasing data coverage, increasing social capital, enhancing support for decision-making, and enhancing the potential for knowledge co-creation [6].

Recently, more and more studies comparing volunteer datasets with professional datasets or with standard methods have shown that citizen scientists generate high-quality reliable data that is comparable to professional data [7]. In fact, successful projects generally rely on a set of methods to improve data accuracy and resolve deviations, including iterative project development, volunteer training and testing, expert verification, replication between volunteers, and statistical modeling of system errors [8]. For example, employing good data validation mechanisms and careful protocol designs for a citizen project can help to improve the quality of volunteer data [9]. Besides, by comparing 63 citizen science projects, Bueno et al. found that the data accuracy of citizen science can meet the requirements of professional researchers, as long as the project has an appropriate scale, number of participants, and participation time [7]. Moreover, citizen science also supports researchers with other options in assessments to explore new methods adopted into their investigating topics. For example, citizen scientists, mainly consisting of local divers from the British Virgin Islands have demonstrated the ability to detect temporal changes of coral reefs in a long-term period of more than 10 years [10].

In Hong Kong, citizen science projects are mostly for public education, while the involvement of citizen scientists in published scientific research is rarely seen. A recently published research about light pollution study in Hong Kong has been a success. The volunteers consisted mainly of high school students who have demonstrated the strength of citizen science in maximizing the coverage of the study and providing reliable and supportive data [11]. However, there is still a huge gap to make citizen science a recognizable monitoring tool in either research or policy-making level in Hong Kong. With the increasing threats to global water security, Hong Kong requires new adaptation and strategy in facing the impairment of local freshwater systems as well as balancing the needs of economic productivity [12]. Citizen science can be an alternative option as one of the new integrated water management strategies which have been unexplored in Hong Kong. While studies focusing on the water quality of the coastal marine environment and the Pearl River Estuary are commonly found and frequently reviewed [13–15], the number of full-scale urban river research seems to be declining. The scarcity of this type of research, especially in the aspect of river quality monitoring, becomes the motivation of this study to develop a framework as a first trial for better adapting such concept at a local level.

This study aims to examine the quality of water data acquired by recruited Citizen Science Leaders (CSLs), who are volunteers with non-scientific background and determine the reliability of the results in comparison to official data from the Environmental Protection Department of Hong Kong (EPD). Despite the fact that the use of volunteer data in water quality monitoring unavoidably exists with errors and bias, this study has proved that it serves well as an initial screening tool in identifying polluted rivers and streams. It has been recommended that giving better testing equipment can overcome such an issue [16]. Thus, in this study, we have used professional water quality testing equipment, and since the volunteers were amateur users when handling such equipment in water quality monitoring, instructed lessons and practical training sessions were given to the volunteers before the study. Exploring the correlation of two datasets may help to gain a better understanding and recognition of citizen science data as an alternative for developing more diverse approaches in future studies of Hong Kong rivers.

## Materials and methods

### Sampling locations

**Fig 1:**
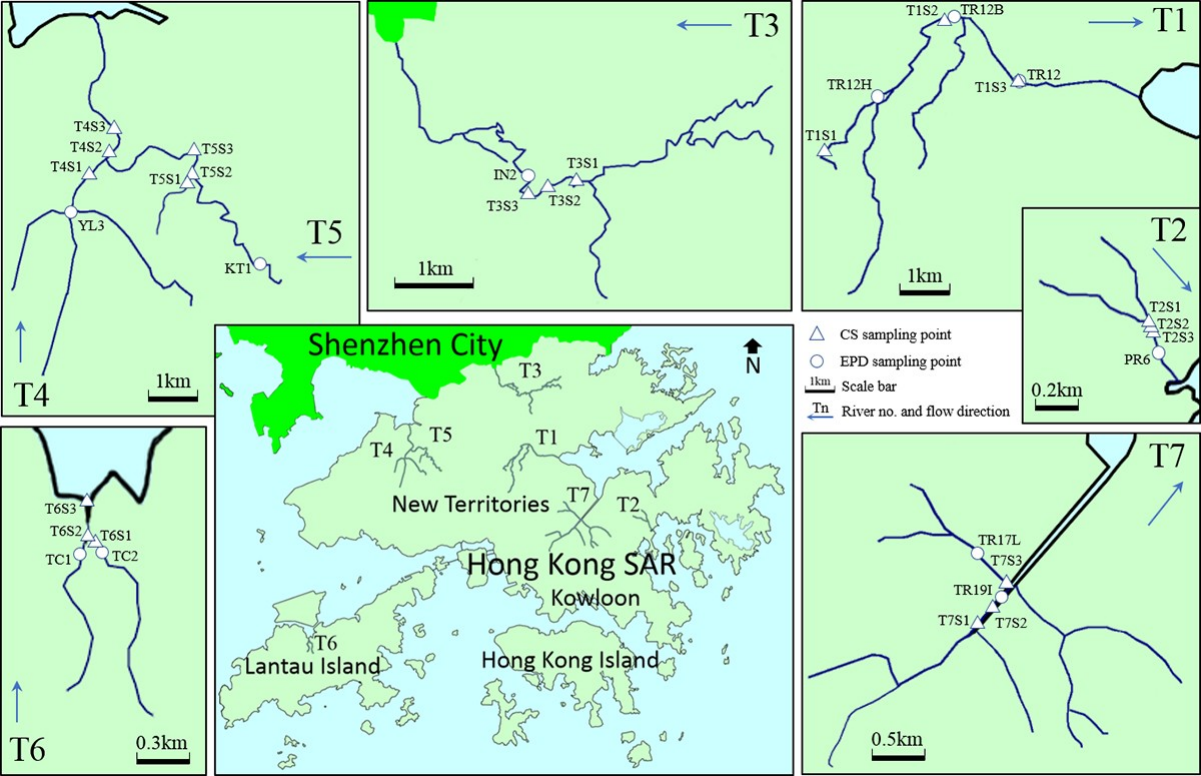
Citizen Science Leader (CSL) and Environmental Protection Department of Hong Kong (EPD) monitoring sites (Δ CSL sampling site; ◯ EPD sampling site). T1: Lam Tsuen River; T2: Sha Kok Mei Stream; T3: River Indus; T4: Yuen Long Creek; T5: Kam Tin River; T6: Tung Chung River; T7: Shing Mun River.

A total of seven streams and rivers of Hong Kong were examined monthly from January 2014 to December 2015 in this study. Six streams and rivers were in the New Territories and one river was on the Lantau Island. They are River Indus (T3), Yuen Long Creek (T4), and Kam Tin River (T5) located in Western New Territories; Lam Tsuen River (T1), Shing Mun River (T7), and Sha Kok Mei Stream (T2) located in Eastern New Territories; Tung Chung River (T6) located on Lantau Island (Fig 1). All of these rivers and streams are isolated from old urban districts as well as from the central business district. Although a few decades ago the surroundings of these locations were considered as rural areas with low population density [17], nowadays most of the rivers and streams are within or adjacent to the new town developed zones. Each river contained three monitoring sites, which were decided by two factors: one is the easy accessibility of citizen scientists reaching the field sites carrying competent equipment; second is the minimum distance from the EPD monitoring sites. All these selected locations were within a distance of 14 to 5180 meters from the official sampling points (Fig 1 and Table 1). All sampling sites were publicly accessible and no permits were required.

**Table 1:** All locations of Citizen Science Leader (CSL) monitoring sites and their separated distances from the nearest Environmental Protection Department of Hong Kong (EPD) monitoring sites (The average discharges at EPD monitoring sites are also showed. NM: not measured).

River / Stream	Locality & Region	CSL site	Paired EPD site		Relative location to the paired EPD site: upstream/downstream	Distance to the nearest EPD site (approximate)
			Label	Average Discharge 2014–2015 (L/s) [18,19]		
Lam Tsuen River	Tai Po, New Territories	T1S1	TR12H	77	Upstream	1600 m
		T1S2	TR12B	137	Upstream	129 m
		T1S3	TR12	55	Upstream	14 m
Sha Kok Mei Stream	Sai Kung, New Territories	T2S1	PR6	NM	Upstream	193 m
		T2S2	PR6	NM	Upstream	154 m
		T2S3	PR6	NM	Upstream	88 m
River Indus	Sheung Shui, New Territories	T3S1	IN2	NM	Upstream	856 m
		T3S2	IN2	NM	Upstream	598 m
		T3S3	IN2	NM	Upstream	298 m
Yuen Long Creek	Yuen Long, New Territories	T4S1	YL3	467	Downstream	707 m
		T4S2	YL3	467	Downstream	1440 m
		T4S3	YL3	467	Downstream	2000 m
Kam Tin River	Yuen Long, New Territories	T5S1	KT1	348	Downstream	4820 m
		T5S2	KT1	348	Downstream	4740 m
		T5S3	KT1	348	Downstream	5180 m
Tung Chung River	Tung Chung, Lantau Island	T6S1	TC2	60	Downstream	26 m
		T6S2	TC1	51	Downstream	44 m
		T6S3	TC1	51	Downstream	322 m
Shing Mun River	Shatin, New Territories	T7S1	TR19I	NM	Upstream	321 m
		T7S2	TR19I	NM	Upstream	49 m
		T7S3	TR17L	NM	Downstream	433 m

### Citizen scientists

Approximately 250 volunteers mainly consisted of the Hongkong and Shanghai Banking Corporation (HSBC) staff participated in this study within two years. They became Citizen Science Leaders (CSLs) and were responsible for all the monthly field measurements and sample collections for at least 3 months without professional supervision on site. Every new participant in this programme was required to attend a whole day of training (including both classroom and on-site training sessions). Each CSL was given a set of training manuals that contained detailed theoretical and practical contents, such as operating manuals for water quality measurement equipment and precautions when measuring water quality. During the training, professional researchers of this project first explained the purpose and significance of the project, and afterwards the definitions of the water quality parameters to be measured and the relationship among them were interpreted. Moreover, researchers explained the detailed procedures, precautions, and techniques of using the monitoring and sampling equipment in such a way that the water quality parameters would be affected. CSLs also took part in field exercises at a river site or a wetland under professional guidance to demonstrate their ability to work independently before completing all training sessions. After the training, a research assistant was responsible for monitoring and coordinating the river investigation activities conducted by the CSLs. Besides, a hotline and an online communication platform were also set up for better communication between the research team and the CSLs.

### The measurements

CSLs who had completed the training were then assigned randomly to one of the seven monitoring teams of seven rivers where they have to obtain measurements and samples from the three designated river sites once a month. On-site water monitoring activity was usually conducted on one of the weekends of each month. The sampling days were not the same as those of the EPD due to the following two reasons: 1. EPD did not disclose to the public the day when their staff would conduct monthly monitoring; 2. CSLs generally had their own full-time jobs and could only participate in this research project on weekends. When CSLs arrived at the monitoring station, they first observed and recorded the local environmental conditions at that time with a camera, and then performed an on-site water quality measurement. The photos and data obtained were instantly transferred to the online database. Physical water parameters including water temperature (°C), pH, conductivity (mS/cm), turbidity (NTU) and dissolved oxygen (mg/L) were selected because they are most commonly used for representing water quality and are relatively less demanding on amateur users with simple testing procedures. Before the CSLs started their monitoring trip, they were given a U-50 Multi-parameter Water Quality Meter (Horiba, Japan) for measuring all the in-situ data. A Van Dorn water sampler was also provided for the sites where sample collection and measurement had to be performed on bridges or high grounds due to restrained accessibility to urbanized river channels. Any necessary calibration of the equipment and/or sterilization of sampling containers was performed in university and the equipment/container was passed over to the CSLs at least one day before their monitoring trip.

For the official data by EPD, their staff visits the sampling sites each month. When EPD staff arrives at the monitoring station, they first check whether their equipment is working properly, and then conduct a series of field observations. The EPD staff use YSI-6820 multi-parameter water quality measurement probes to perform on-site measurements of temperature, pH, turbidity, and dissolved oxygen and record the values in the data logger. The readings obtained are then compared with the normal range of the monitoring station, and if any abnormal readings are observed, they repeat the measurements to confirm the validity of the initial readings. All the data and other on-site observations are initially recorded on a portable PC, which is later downloaded and entered into the EPD monitoring database in the EPD office. The database was specially designed for effective data storage, management, and analysis [20].

### Data analysis

The in-situ official data for comparison were obtained from EPD online database [18,19]. When using the official data as a reference to make comparisons with the volunteer data, several assumptions were made: 1) Difference in measurement results caused by the difference in measuring instruments is considered to be non-significant. 2) The noises produced by the temporal variance of CSL and EPD sampling points were constrained through a continuous 2-year monitoring period.

Since the majority of the data was heavily tailed and failed to comply with normal distribution due to inherent bias and possible contamination issues within the testing samples, we choose medians to define the central value of both datasets and to determine their differences between volunteer data and official data [21]. The percentage difference in the median was calculated for each parameter to assess the precision of volunteer data and to check whether the data is in agreement with the official data, reflecting the same phenomenon of rivers in Hong Kong. Two non-parametric tests were also performed in IBM SPSS Statistics ver.22. Mann-Whitney U test was used for testing whether CSL and EPD data came from the same population of each river, and possibly had significant differences in their medians; Spearman's Rank-Order Correlation was used for interpreting the reliability of detecting the changes in water quality among the 7 rivers in longer-term based on their rank changing in the amount of dissolved oxygen and the turbidity.

## Results

### Water temperature

**Fig 2:**
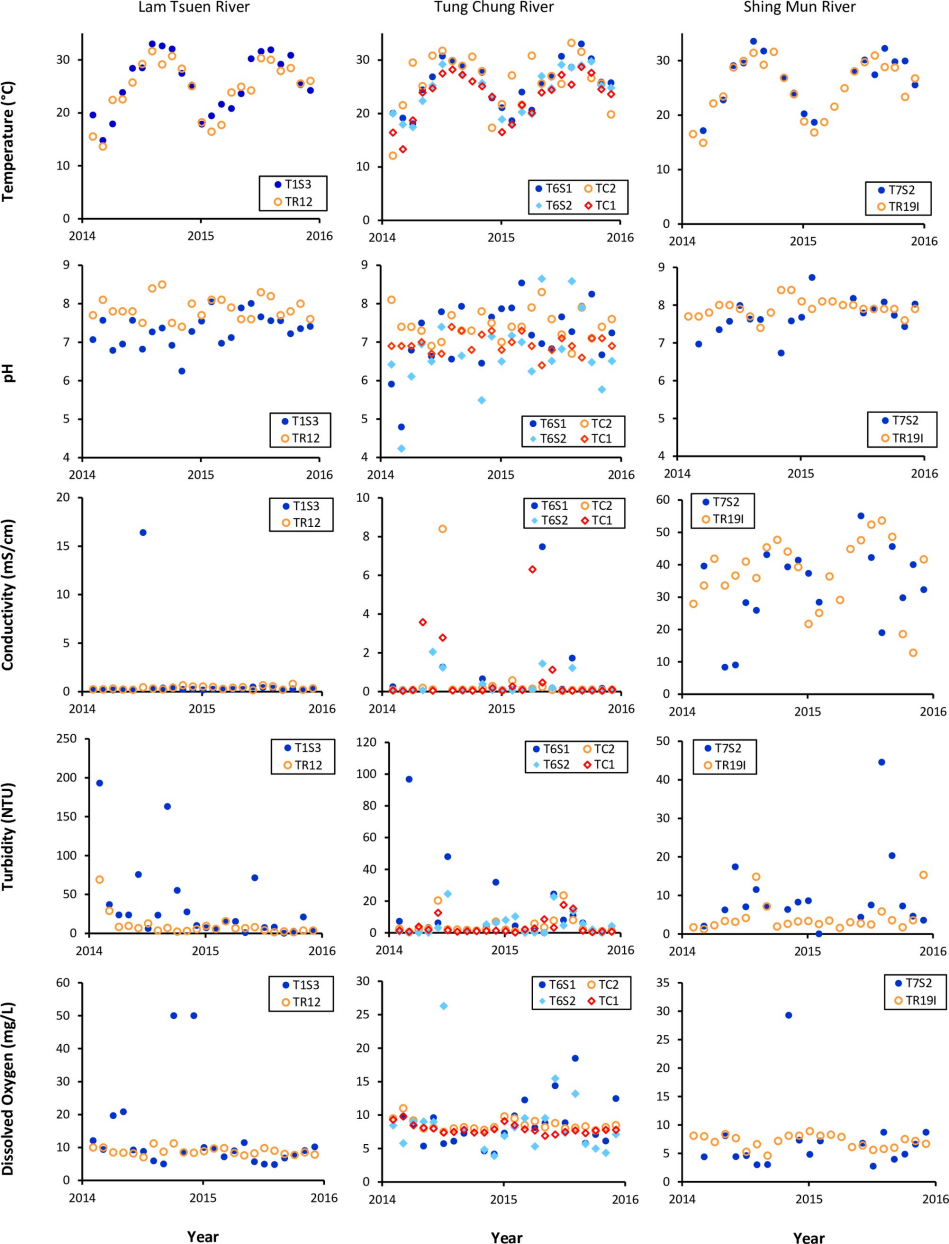
Scatter plots of five water parameters measured by Citizen Science Leaders (CSLs) and the Environmental Protection Department of Hong Kong (EPD) between 2014 and 2015. The CSL monitoring sites (represented by solid-filled symbols, ● ◆) were 50 m or less apart from EPD monitoring sites (represented by hollow symbols, ○ ◇).

**Fig 3:**
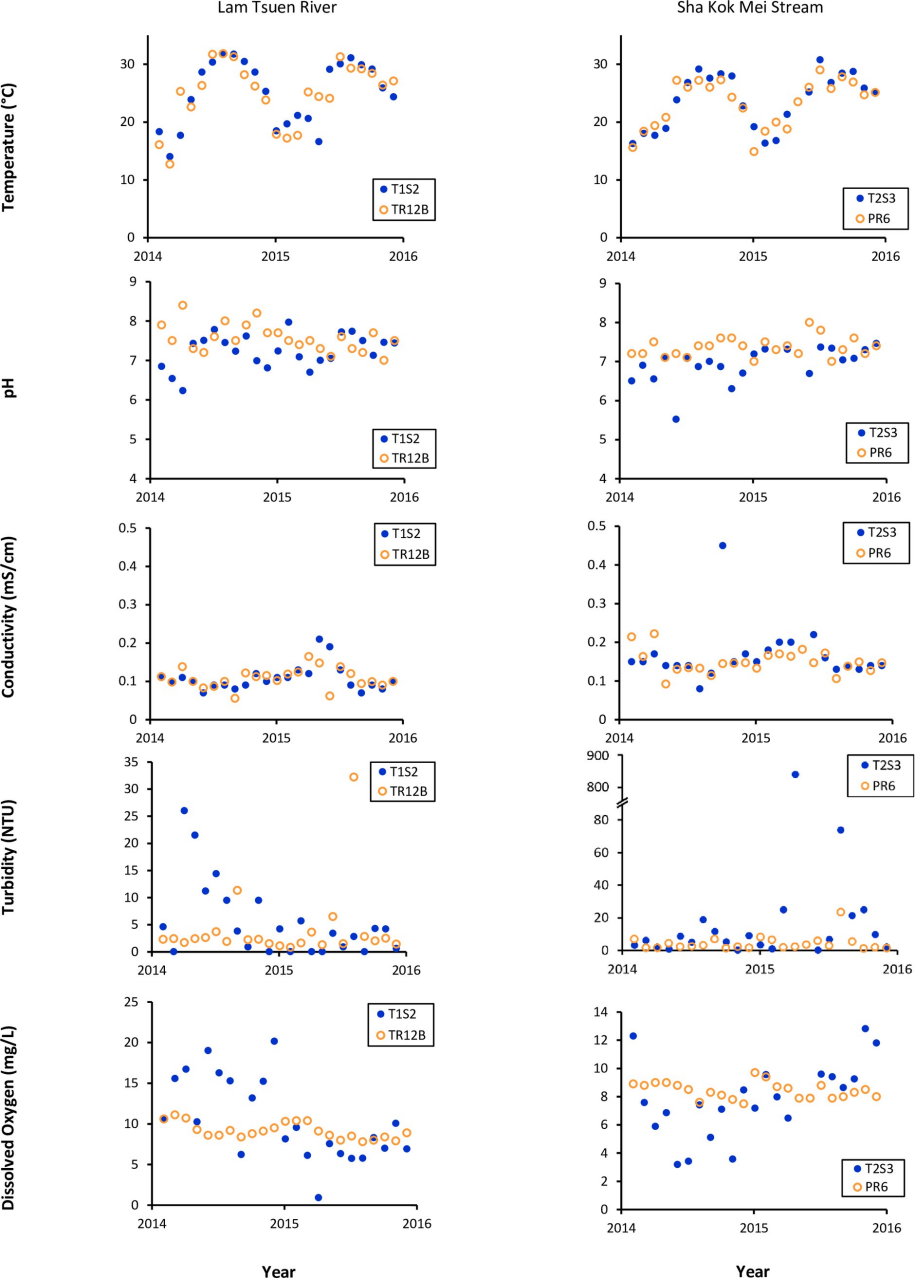
Scatter plots of five water parameters measured by Citizen Science Leaders (CSLs) and the Environmental Protection Department of Hong Kong (EPD) between 2014 and 2015. The CSL monitoring sites (represented by solid-filled symbols, ●) were about 100 m apart from EPD monitoring sites (represented by hollow symbols, ○).

Among the five water quality parameters, water temperature measured by CSL has the highest degree of coincidence with the EPD data. The overall CSL datasets from various rivers were closely correlated to official data measured by EPD with minimum spatial variation (less than 100 m apart from each other). In comparison to their medians, the CSL data had a similar range to official data, from 25.24°C to 27.71°C while the percentage difference between both medians was less than 8% (Tables 2 and 3). The 2-year measurement in Lam Tsuen River performed by CSLs turned out to have the smallest median difference with EPD data, as low as 0.55% and 0.04% in two representing sites (Tables 2 and 3). Statistically, there were also no significant differences between the distribution of volunteer data and official data (p-value ≥ 0.05) for all the paired sites as close as 100 m apart or less (Tables 2 and 3). Both CSL and official data clearly displayed the same seasonal patterns of water temperature having the peak measurement in summer months while the lowest in winter (Figs 2 and 3). Regardless of CSL or EPD data, the given temperature measurements existed in all seven rivers with similar regularity and consistency.

**Table 2:** The sample size (n), median and interquartile range (IQR) of the 2-year measurements taken by Citizen Science Leaders (CSLs) compared with those taken by the Environmental Protection Department of Hong Kong (EPD) where two locations were less than 50 m apart from each other.

		Volunteer data—CSLs				Official data—EPD				% different between both medians	p-value
Parameter	River	Sampling site	n	median	IQR	Sampling site	n	median	IQR		
Water temperature (°C)	Lam Tsuen River	T1S3	24	25.24	10.44	TR12	24	25.25	9.23	-0.04	0.496*
	Tung Chung River	T6S1	23	25.74	8.26	TC2	24	26.8	8.78	-4.04	0.349*
	Tung Chung River	T6S2	22	24.735	7.93	TC1	24	24.15	7.85	2.39	0.468*
	Shing Mun River	T7S2	18	27.71	7.68	TR19I	24	25.8	7.6	7.14	0.269*
pH	Lam Tsuen River	T1S3	24	7.315	0.57	TR12	24	7.8	0.47	-6.42	(<0.005)
	Tung Chung River	T6S1	23	7.27	1.22	TC2	24	7.4	0.47	-1.77	0.53*
	Tung Chung River	T6S2	22	6.51	0.78	TC1	24	6.9	0.38	-5.82	0.046
	Shing Mun River	T7S2	18	7.655	0.46	TR19I	24	7.9	0.2	-3.15	0.037
Conductivity (mS/cm)	Lam Tsuen River	T1S3	24	0.23	0.195	TR12	24	0.3295	0.276	-35.57	0.021
	Tung Chung River	T6S1	23	0.12	0.08	TC2	24	0.0935	0.084	24.82	0.197*
	Tung Chung River	T6S2	22	0.08	0.162	TC1	24	0.064	0.191	22.22	0.261*
	Shing Mun River	T7S2	18	34.8	17.425	TR19I	24	37.95	17.034	-8.66	0.274*
Turbidity (NTU)	Lam Tsuen River	T1S3	24	15.3	28.9	TR12	24	5.85	5.85	89.36	0.019
	Tung Chung River	T6S1	23	3.17	6.3	TC2	24	1.95	3.9	47.66	0.198*
	Tung Chung River	T6S2	22	2.565	6.8	TC1	24	1.3	2.35	65.46	0.495*
	Shing Mun River	T7S2	18	7.15	5.55	TR19I	24	3.05	1.45	80.39	0.001
DO (mg/L)	Lam Tsuen River	T1S3	24	9.04	4.28	TR12	24	8.5	1.58	6.16	0.726*
	Tung Chung River	T6S1	23	8.14	3.71	TC2	24	8.2	1.17	-0.73	0.482*
	Tung Chung River	T6S2	22	7.585	3.57	TC1	24	7.8	0.97	-2.79	0.495*
	Shing Mun River	T7S2	18	5.73	3.98	TR19I	24	7.35	1.92	-24.77	0.124*

Their median differences are shown in percentage and p-values are confirmed whether they came from the same population by using Mann–Whitney U test.

* The CSL data is not significantly different to the EPD data (p-value ≥ 0.05)

**Table 3:** The sample size (n), median and interquartile range (IQR) of the 2-year measurements taken by Citizen Science Leaders (CSLs) compared with those taken by the Environmental Protection Department of Hong Kong (EPD) where two locations were about 100 m apart from each other.

		Volunteer data—CSLs				Official data—EPD				% different between both medians	p-value
Parameter	River	Sampling site	n	median	IQR	Sampling site	n	median	IQR		
Water temperature (°C)	Lam Tsuen River	T1S2	24	25.61	10.1	TR12B	24	25.75	8.95	-0.55	0.805*
	Sha Kok Mei Stream	T2S3	23	25.04	9.92	PR6	24	24.5	7.13	2.18	0.733*
pH	Lam Tsuen River	T1S2	24	7.335	0.52	TR12B	24	7.5	0.4	-2.22	0.025
	Sha Kok Mei Stream	T2S3	23	7.04	0.62	PR6	24	7.4	0.3	-4.99	(<0.005)
Conductivity (mS/cm)	Lam Tsuen River	T1S2	24	0.1	0.03	TR12B	24	0.101	0.031	-1.00	0.86*
	Sha Kok Mei Stream	T2S3	23	0.15	0.03	PR6	24	0.147	0.033	2.02	0.436*
Turbidity (NTU)	Lam Tsuen River	T1S2	24	4	8.63	TR12B	24	2.25	1.25	56.00	0.464*
	Sha Kok Mei Stream	T2S3	23	5.87	16.87	PR6	24	2.2	4.2	90.95	0.053*
DO (mg/L)	Lam Tsuen River	T1S2	24	8.935	8.77	TR12B	24	9	1.8	-0.72	0.789*
	Sha Kok Mei Stream	T2S3	22	7.495	3.32	PR6	24	8.5	0.95	-12.57	0.129*

Their median differences are shown in percentage and p-values are confirmed whether they came from the same population by using Mann–Whitney U test.

* The measurements of CSL are no significantly different to the measurements of EPD (p-value ≥ 0.05)

### pH

The pH measurement appeared to be with more variability and less consistency conducted by CSL than that by EPD. For the EPD’s dataset, most of the measurements throughout the 2-year monitoring varied about 1 pH value of difference at maximum. Although some rivers such as Tung Chung River had slightly larger variability, the EPD measurements were fairly consistent around one particular median level for each river (Figs 2 and 3). On the other hand, large fluctuations were commonly observed in the pH data collected by CSLs. Obvious outliers were spreading across the whole sampling period (Figs 2 and 3). For most of theriver datasets, CSL measurements observed to be lower than the EPD figures (Figs 2 and 3). For example, the pH value for the Tung Chung River was recorded in the range from 4.23 to 8.65 by CSL while it was recorded in the range of 6.4 to 8.3 by EPD (Fig 2). Hence, the pH data collected by CSL and EPD partially overlapped with each other during the period from 2014 to 2015. The distributions of the two datasets proved to be statistically different (p-value < 0.05) in most of the paired cases, whereas the percentage differences of their medians were under 6.5% (Table 2).

### Conductivity

Conductivity was another parameter showing a comparable result of CSL data and EPD data. For those rivers and streams with less tidal influence, both datasets indicated steady changes in ionic contents of all water samples despite occasional outliers recorded by CSLs. The sampling sites of Lam Tsuen River and Sha Kok Mei Stream were set away from the river mouths. Their typical conductivity levels were around 0.1 mS/cm and 0.17 mS/cm respectively (Fig 3). Shing Mun River, by contrast, has been heavily influenced by the tidal events. Its conductivity levels measured by CSLs and EPD were swinging between 9 mS/cm and 53 mS/cm over the months (Fig 2). The median conductivity levels measured in Shing Mun River by CSLs and EPD were 35mS/cm and 37mS/cm respectively (Table 2), which were nearly 100 times higher than those measured in other rivers and streams without tidal influence. In terms of percentage difference, some paired sites with low levels of conductivity (e.g. Tung Chung River and Lam Tsuen River) generally consisted of 22.2% to 35.6% difference between CSL and EPD data (Table 2), while some pairs such as from Sha Kok Mei Stream were better correlated with less than 2% of the median difference (Table 3). In addition, the distributions between CSL and EPD data of conductivity measurement were statistically shown with no significant difference for five out of six pairs of sites whose separated distances were under 100 m (p-value ≥ 0.05) (Tables 2 and 3).

### Turbidity

**Fig 4:**
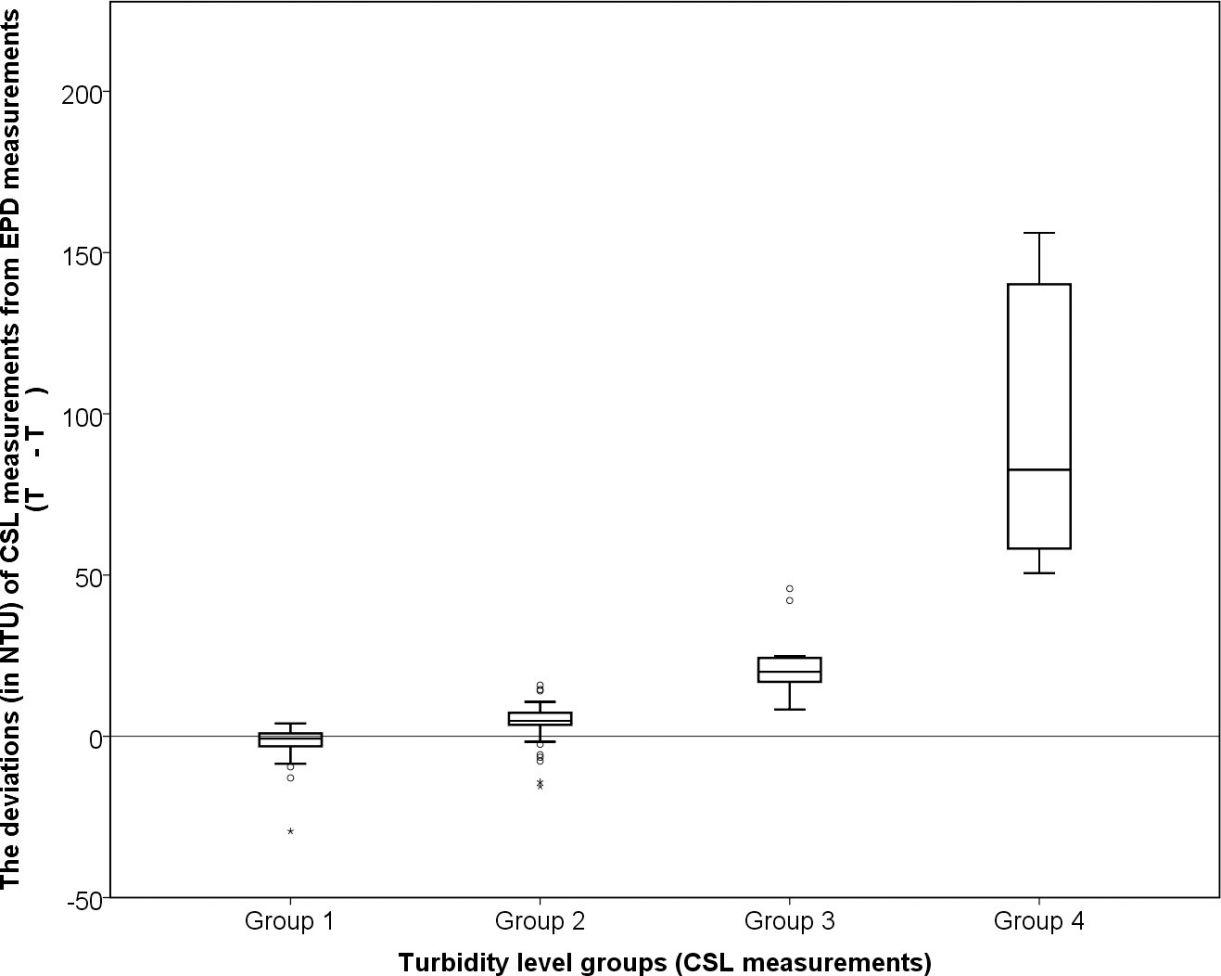
A box plot showing the deviation between the CSL-measured turbidity level (T_CSL_) and the EPD-measured turbidity level (T_EPD_) on all the paired monitoring sites with less than 100 m separated distances. Each interval group contains the actual CSL measurements within a specific turbidity range: Group 1—Turbidity ≦5.5 NTU; Group 2–5.5 NTU < Turbidity ≦20.5 NTU; Group 3–20.5 NTU < Turbidity ≦50.5 NTU; Group 4–50.5 NTU < Turbidity.

The measurement results of turbidity showed that there were stronger correlations between CSL data and EPD data when measuring the rivers and streams with less suspended solid loads. In general, the changing trend of CSL data highly corresponded to those of EPD data when the turbidity level retained below 20 NTU (Figs 2 & 3). However, CSLs tended to take more overestimated measurements from high turbid samples, which were indicated by large outliers throughout the sampling period. The differences between CSL and EPD pairs were also exponentially increasing with exceptional turbidity levels (Fig 4). As a result, the percentage difference of medians between two datasets could reach as high as around 90%, especially for the paired sites in Sha Kok Mei Stream and Lam Tsuen River (Tables 2 & 3). In contrast, the paired sites in mid-stream Lam Tsuen River (Table 3) and Sha Kok Mei Stream (data not showed) had about 40 to 60% of median differences, which were still distinguishably high compared with other parameters. In spite of the disparate association of two datasets at various levels of turbidity, their overall distributions both actually resembled in terms of mean rank. Four out of six pairs of site data were determined to have no statistically significant difference between the CSL data and EPD data (p-value ≥ 0.05), which revealed that those pairs even with large differences in medians were considered as statistically "similar" within the same population.

### Dissolved oxygen

There were overall strong correlations between CSL and EPD measurements of dissolved oxygen, which were sometimes demoted by the factor of geographical bias between the sampling sites of CSL and those of EPD. With the separated distance below 50 m, the CSL measurements responded adequately to the yearly trend of dissolved oxygen measured by EPD (Fig 2). The CSL data, except the Shing Mun River, contained less than 6.2% of the difference in medians to the EPD data (Table 2). On the other hand, slightly weaken correlations were frequently observed from the site pairs with more than 100 m of separation of where EPD measurements were conducted (data not showed). These unmatched pairs were probably determined by larger unknown variations existing between the sites, as well as the rising amount of invalid dissolved oxygen data from some particular monitoring sites, such as in mid-stream Lam Tsuen River and Sha Kok Mei Stream indicated by the extreme outliers of CSL measurements. It is worth noting that the medians between CSL and EPD data were pretty similar with only less than 13% of differences, except the middle stream of Shing Mun River. Due to the resembling patterns, the distribution of two datasets had no statistically significant difference in terms of dissolved oxygen measurement, which were indicated by uplifted p-value (equal or greater than 0.05) among all the site pairs with separated distance below 100 m (Tables 2 & 3).

**Fig 5:**
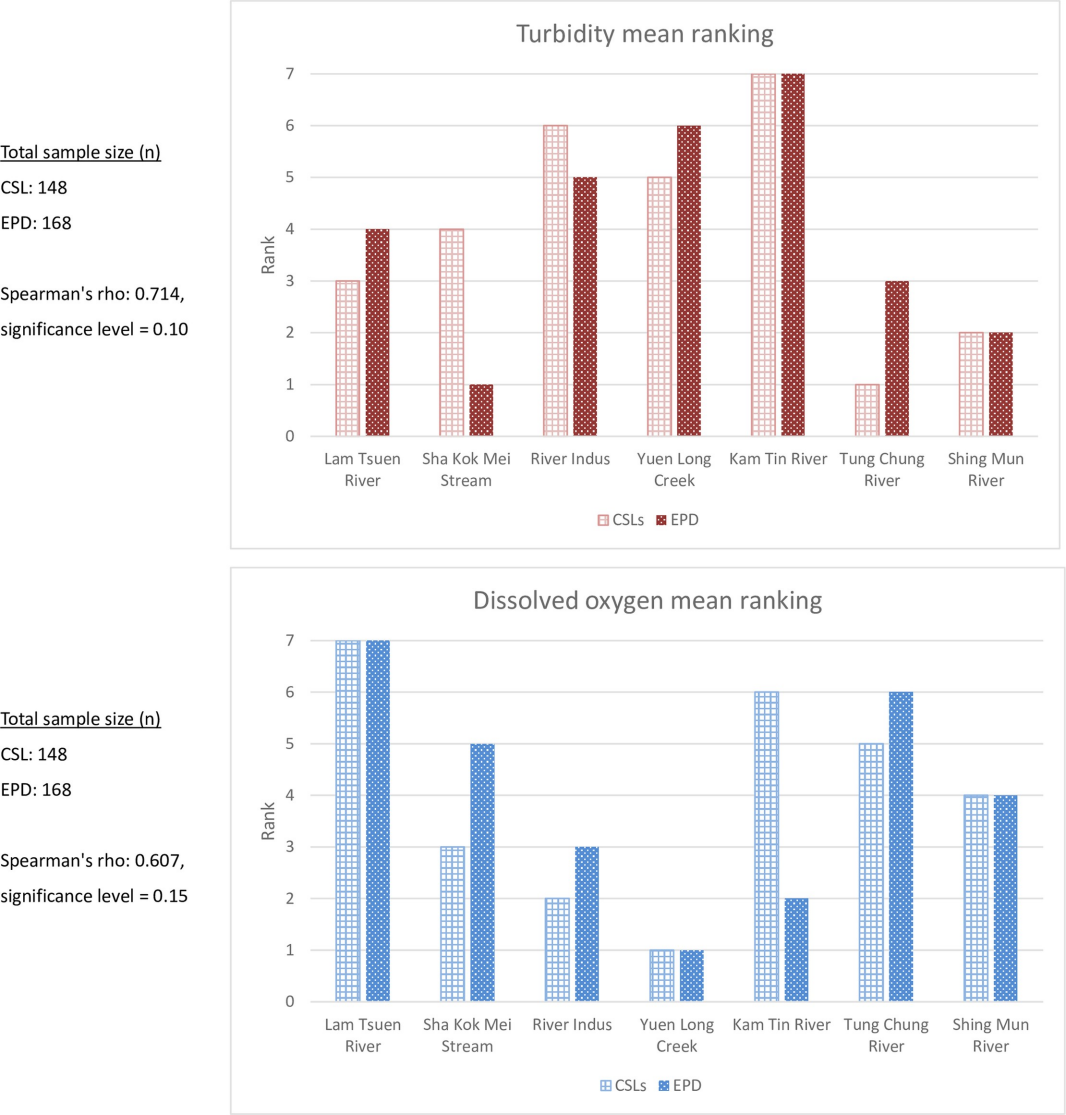
The 2-year mean ranking of 7 rivers in terms of turbidity and dissolved oxygen according to the Citizen Science Leader (CSL) and the Environmental Protection Department of Hong Kong (EPD) data separately. The river ranking in turbidity is increased with more turbid water on an average of 24 months. The river ranking in dissolved oxygen is increased with a higher oxygen level in the water on an average of 24 months.

In this study, a ranking of turbidity and dissolved oxygen levels were respectively given to each river according to its 2-year means and the ranking orders of 7 rivers with CSL data have been found to be similar to those with EPD data (Fig 5). Tung Chung River, for instance, obtained lower ranks from both datasets in terms of turbidity, while both its CSL and EPD mean ranks of dissolved oxygen levels are among the second and the third highest river respectively. In contrast, River Indus, Yuen Long Creek, and Kam Tin River are three rivers located in the northern part of Western New Territories in Hong Kong, where untreated domestic discharge and agricultural runoff have always been persistent water issues [22,23]. These rivers received a low ranking in dissolved oxygen and high ranking in turbidity from both official and volunteer data. The strength of the correlation was further confirmed by the results of Spearman's rank statistic test. The results showed that the Spearman's correlation coefficient of the two turbidity datasets was 0.714 and a strong linear relationship was confirmed at a significance level of 0.1, while the Spearman's correlation coefficient of two dissolved oxygen datasets was 0.607 and a moderate positive relationship was confirmed at a significance level of 0.15.

**Fig 6:**
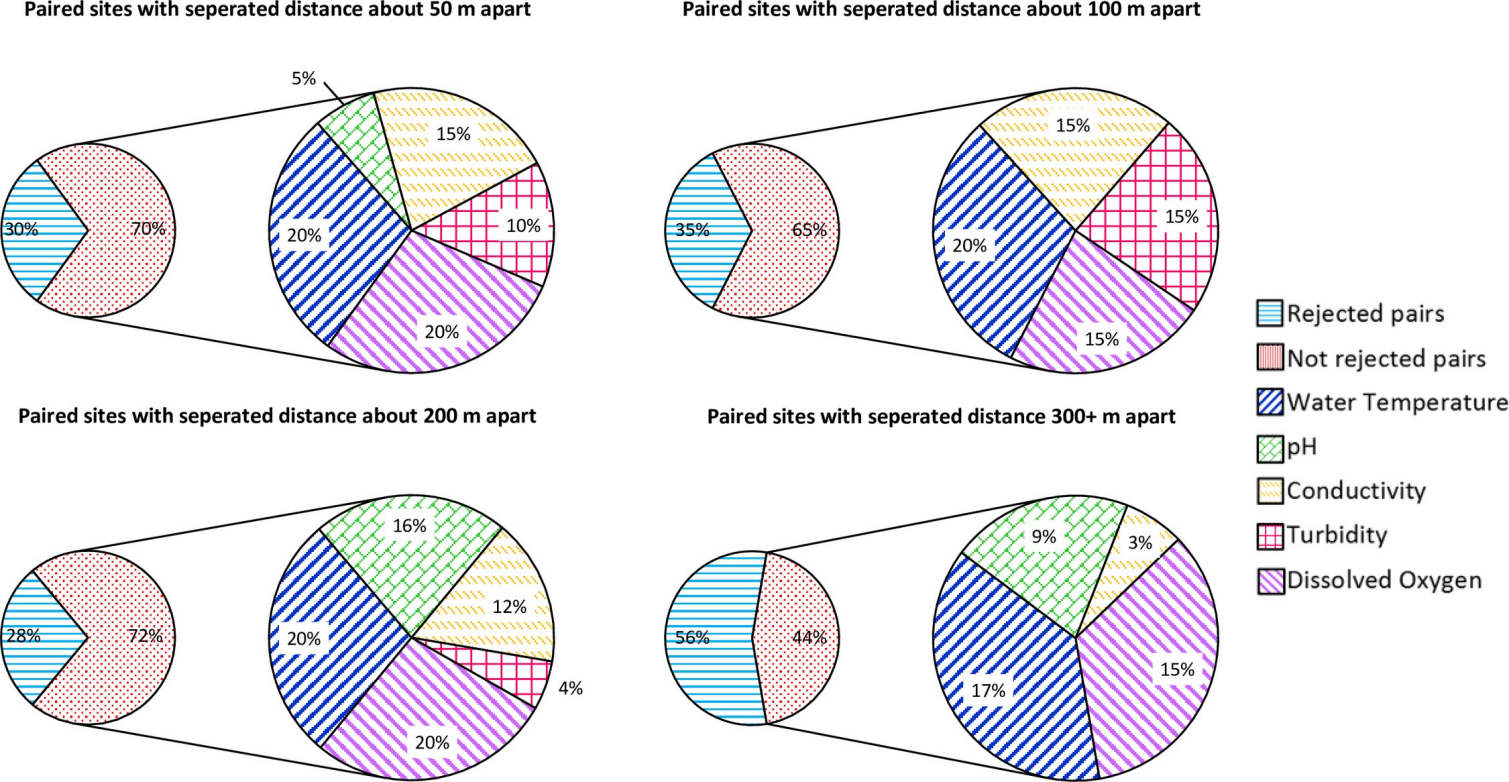
The overall results in percentage of Mann-Whitney U test on all Citizen Science Leader (CSL) site data paired with the Environmental Protection Department of Hong Kong (EPD) site data from a total of 7 rivers. H_0_—The distribution of CSL data is no different from the distribution of EPD data. H_a_—The distribution of CSL data is different from the distribution of EPD data. The types of parameter within not rejected group are also shown in percentage.

Mann-Whitney U test was conducted to test the hypothesis that the volunteer data and the official data pairs are coming from the same section. The rejected data pairs show the null hypothesis is against with strong significance (p-value < 0.05) while the not rejected data pairs are failing to reject the null hypothesis (p-value ≥ 0.05). The result revealed that their differences in sampling locations of the same river were rejecting the possibility if the separated distance of two monitoring locations was distinct, i.e. > 300 m (Fig 6). The rejected pairs (p-value < 0.05), which were statistically proved to have significantly different in their representing populations, were about 20% to 30% of the total site pairs within 100 m distance apart from each other. About 200 m of separation, the number remained steadily close with 28% of the data pairs considered as discrete distributions. Although over half of the data pairs with above 300 m of separated distance were found to be predominantly different, at least 65% of them, below 300 m of separated distances from EPD sites, were tested with matching distributions.

## Discussion

The performance of citizen scientists in this project effectively demonstrates their reliable participation in water monitoring activities in Hong Kong. The way CSLs acquired water temperature data confirms a compatible result with what the EPD does. The identical patterns of both volunteer and official data indicate the dominant factor of seasons that primarily determine the heat retained in streams and rivers instead of other thermal sources. This is also supported by the statistical test results, with the inability of the given all kinds of site separation reducing the correlated magnitude of volunteer and official data in terms of water temperature measurement. Besides, the level of conductivity measured by CSLs generally achieved a presumptive result of the EPD measurements. By referring to EPD reports [13–15], the CSL measurements of conductivity respectably matched the data of total dissolved solids present in different rivers. In fact, various salinity levels of different streams and rivers can be accurately distinguished by interpreting the volunteer data.

Nonetheless, the volunteer data acquired by CSLs clearly exhibited more randomness than the official data acquired by EPD staff causing less overall precision. Only 5% or less of the CSL measurements on water pH was tested not significantly different from the EPD measurements, where the values were measured within 100 m of separated distance. Moreover, the CSLs with basic sampling techniques and limited water quality monitoring experience proved insufficient for handing some urbanized rivers in Hong Kong. Since occasional highly turbid water conditions and dynamic water depth were often present in forms of the drainage channel, more adaptable skills were required to collect the water samples from such rivers using a Van Dorn water sampler. When the sampler was near to a deeper water level, the sediments from the drainage bottom could possibly be collected together with the water sample, which potentially can lead to some overestimations of the turbidity level. This is one of the reasons explaining the CSLs measurements of low turbidity levels that are closely correlated to EPD measurements instead of high turbidity levels. Unlike turbidity, the effect of bottom sediments was less evident on the conductivity measurements, indicating that conductivity is a more robust parameter. Another reason is that some critical measuring procedures were to be followed when using the professional devices, which some of the CSLs found very technical to follow. For example, the water quality meter used in this study required a steady vertical movement of the DO sensor along the water column and at least 10 minutes of waiting time for obtaining the stabilized reading, and if the guidelines were not properly followed, the dissolved oxygen measurement will be either underestimated or overestimated. Based on the logging time of the device, it was seen that the average measuring time of CSLs was around 5 minutes or less for each measurement, which is fairly less than what was stipulated in the guidelines. General feedback from the CSLs further indicated that they simply forgot or overlooked some minor steps or guidelines mentioned to them in the training. This led to the increased variability in the overall dataset reflected by various skill levels of the CSL groups. Such inconsistency is, however, understandable given that there was usually a gap period from the completion of their training to their first day of monitoring work, as well as having different expectations on unrestricted working hours spent each time by different CSL groups. While the volunteer data might not be as good as professional data in terms of accuracy of figures, the capability of citizen scientists in determining long-term changes in water conditions in various rivers and streams in Hong Kong has shown practical values in this programme.

Besides, the level of certain parameters (such as turbidity, and/or dissolved oxygen in the absence of obvious sources of thermal pollution) is sufficient to indicate the overall water quality of a river. Therefore, the quality of a river can be simply ranked based on the order of these parameters in many rivers. Herein, this can be reflected from the ranking of river water quality, which was based on turbidity and dissolved oxygen levels, as well as on the results of Spearman's rank statistical test. This demonstrates the potential of using volunteer data for effectively distinguishing rivers with "good" or "bad" water quality in a more illustrative way.

The possibility of substituting volunteer data for the missing official data in future analyses is determined by whether both the data are coming from the same section. According to the results of Mann-Whitney U test, minimal sampling location distance is suggested to keep the volunteer data more comparable to the official data when adopting both datasets in one analysis. Besides, the location factors causing adaptable issues should be taken into consideration, especially for some water quality parameters that can be easily influenced by the unknown sources of water discharge. According to Mann-Whitney U test results, the overall rejected percentages of conductivity and turbidity data pairs were increasing with increasing separated distance between the two monitoring locations. This shows that the distinct geographical differences make volunteer data more unlikely to correlate with official data. Nonetheless, both water temperature and dissolved oxygen data seemed resistant to such spatial variance.

This study recruited a large number of volunteers, of which almost all were laymen to water quality monitoring. The volunteers were entitled CSLs after a systematic classroom and field training and were then assigned to the designated rivers or streams for water quality monitoring. In this study, we used high precision portable water quality monitoring instruments that are commonly used in scientific research institutions instead of using simple testing kits with high inaccuracy and low resolution. The main purpose was to test whether CSLs with advanced experimental instruments could provide results with high reliability. It should be noted that, since the volunteers had their own full-time jobs elsewhere, they could only use limited spare time to participate in this research, thus, this research project was not intended to work with specific sampling times and sampling points. The point-to-point comparison of EPD data is not feasible in actual operation, so this study used long-term monitoring with the mean of different data points to analyze the water quality. Since this project describes the quality of the river water in Hong Kong, and identify which section of the river is polluted, it was generally considered successful as a preliminary discrimination test. Professionals can conduct more detailed and professional monitoring and analysis by using the volunteer data. Due to the limited manpower and resources, the government often overlooks some sampling points. CSLs with sufficient man powers can obtain a large amount of data, which is a critical first step for more in-depth research.

By comparison with official data, the five selected water quality parameters measured by citizen scientists can reflect the overall pollution status of the rivers in Hong Kong. Among these, the measurement accuracy of dissolved oxygen largely depends on the degree to which citizen scientists have mastered the specifications for the use of water quality test equipment and their compliance in implementation. The measurement accuracy of turbidity depends on the condition of the river channel and the CSLs' skill of using Van Dorn water sampler. For instance, when there are a lot of sediments at the bottom of any river and the water is shallow, there would be a high chance of collecting the lifted-up sediments from the bottom with the Van Dorn sampler, especially when the sampler (CSLs) has amateur skills. Relatively, water temperature and conductivity are more robust parameters that remain least affected by the sediments that might be collected at the bottom. In addition, we believe that the measurement accuracy of the pH is not related to the measuring skills of CSLs rather it is related to the nature of water, which is highly dependent on the type and concentration of dissolved substances in certain sections of the river. These findings can surely provide very useful information to similar citizen scientist projects in the future.

## Conclusions

This study demonstrates a successful outcome of utilizing citizen science programme in an urban river monitoring. A local research framework has been developed for hundreds of HSBC volunteers participating in a regular water quality monitoring of 7 rivers and streams in Hong Kong. Certainly, there is still much room for improvement in the project framework in order for it to become a reference model for future citizen science projects in Hong Kong. We recommend keeping the scale of the future project as large as possible and to extend the study length for at least 2 years to be considered effective in overcoming the uncertainty brought by citizen science data. Inviting more community stakeholders to join nearby monitoring activities can further enhance the input of citizen science programme in terms of their length of engagement and acquired data quality. Regarding hundreds of rivers and streams in Hong Kong that lacks the coverage of regular water quality monitoring, we deem citizen science to be an appropriate and valuable tool in safeguarding the freshwater ecosystem against the contaminations due to rapid urban land expansion in Hong Kong.

This study also revealed the capability of citizen scientists in detecting long-term changes in river water quality. The results of water temperature and conductivity data were highly comparable to the official data from the government authority. Moderate to strong correlations in water pH, turbidity, and dissolved oxygen levels between citizen scientists and the official data. Our analysis suggests that citizen scientists are capable of handling the monitoring tasks of small and polluted rivers. When encountering rivers with complex hydrological conditions or high levels of pollution, enhanced sampling skills training and detailed explanations will help improve the accuracy of water quality monitoring data.

## References

[1] Boylen CW, Howe EA, Bartkowski JS, Eichler LW. Augmentation of a Long-term Monitoring Program for Lake George, NY by Citizen Volunteers. *Lake Reserv Manage*. 2004 6;20(2):121–9.

[2] Weyhenmeyer GA, Mackay M, Stockwell JD, Thiery W, Grossart H, Augusto-Silva PB, et al Citizen science shows systematic changes in the temperature difference between air and inland waters with global warming. *Sc*. *Rep*. 2017 3 6;7. 10.1038/srep43890 PMC5338347 28262715

[3] Abbott BW, Moatar F, Gauthier O, Fovet O, Antoine V, Ragueneau O. Trends and seasonality of river nutrients in agricultural catchments: 18 years of weekly citizen science in France. *Sci Total Environ*. 2018;624:845–858. 10.1016/j.scitotenv.2017.12.176 29274609

[4] McGoff E, Dunn F, Cachazo LM, Williams P, Biggs J, Nicolet P, et al Finding clean water habitats in urban landscapes: professional researcher vs citizen science approaches. *Sci*. *Total Environ*. 2017;581:105–116. 10.1016/j.scitotenv.2016.11.215 28069307

[5] Edwards PM, Shaloum G, Bedell D. A unique role for citizen science in ecological restoration: a case study in streams. *Restor*. *Ecol*. 2018;26:29–35.

[6] Njue N, Stenfert Kroese J, Gräf J, Jacobs SR, Weeser B, Breuer L et al Citizen science in hydrological monitoring and ecosystem services management: State of the art and future prospects. *Sci Total Environ*. 2019;693:133531 10.1016/j.scitotenv.2019.07.337 31635016

[7] Aceves-Bueno E, Adeleye A, Feraud M, Huang Y, Tao M, Yang Y, et al The accuracy of citizen science data: a quantitative review. *Bull Ecol Soc Am*. 2017;98(4):278–290.

[8] Kosmala M, Wiggins A, Swanson A, Simmons B. Assessing data quality in citizen science. *Front Ecol Environ*. 2016;14:551–560.

[9] Wiggins A, Newman G, Stevenson RD, Crowston K. Mechanisms for data quality and validation in citizen science. Proceedings of 2011 IEEE Seventh International Conference on e-Science Workshops; 2011 Dec 5–8; Stockholm, Sweden. IEEE; 2011.

[10] Forrester G, Baily P, Conetta D, Forrester L, Kintzing E, Jarecki L. Comparing monitoring data collected by volunteers and professionals shows that citizen scientists can detect long-term change on coral reefs. *J Nat Conserv*. 2015 4;24:1–9.

[11] Pun CSJ, So CW. Night-sky brightness monitoring in Hong Kong. *Environ Monit Assess*. 2012;184: 2537–57. 10.1007/s10661-011-2136-1 21713499

[12] Vörösmarty CJ, McIntyre PB, Gessner MO, Dudgeon D, Prusevich A, Green P, et al Global threats to human water security and river biodiversity. *Nature* 2010;467: 555–61. 10.1038/nature09440 20882010

[13] Tsang YY, Mak CW, Liebich C, Lam SW, Sze ET, Chan KM. Microplastic pollution in the marine waters and sediments of Hong Kong. *Mar Pollut Bull*. 2017; 115(1): 20–28. 27939688 10.1016/j.marpolbul.2016.11.003

[14] Zhou T, Wu J, Peng S. Assessing the effects of landscape pattern on river water quality at multiple scales: A case study of the Dongjiang River watershed, China. *Ecol Indic*. 2012; 23: 166–75.

[15] Chen X, Li YS, Liu Z, Yin K, Li Z, Wai OWH, et al Integration of multi-source data for water quality classification in the Pearl River estuary and its adjacent coastal waters of Hong Kong. *Cont Shelf Res*. 2004;24(16):1827–43.

[16] Loperfido JV, Beyer P, Just CL, Schnoor JL. Uses and Biases of Volunteer Water Quality Data. *Environ Sci Technol*. 2010 10 1; 44(19):7193–99. 10.1021/es100164c 20540530

[17] Hui ECM, Lam MCM. A study of commuting patterns of new town residents in Hong Kong. *Habitat Int*. 2005 9;29(3):421–437.

[18] [EPD] Environmental Protection Department of Hong Kong. River Water Quality in Hong Kong in 2014 [Internet]. 2015 [Cited 2019 August 24]. Available from: http://www. https://www.epd.gov.hk/epd/english/environmentinhk/water/hkwqrc/waterquality/river-2.html.

[19] [EPD] Environmental Protection Department of Hong Kong. River Water Quality in Hong Kong in 2015 [Internet]. 2016 [Cited 2019 August 24]. Available from: http://www.epd.gov.hk/epd/english/environmentinhk/water/hkwqrc/waterquality/river-2.html.

[20] [EPD] Environmental Protection Department of Hong Kong. 20 Years of River Water Quality Monitoring in Hong Kong, 1986–2005. [Internet]. 2006 [Cited 2019 August 24]. Available from: https://www.epd.gov.hk/epd/misc/river_quality/1986-2005/eng/director_menu.htm.

[21] Jollymore A, Haines MJ, Satterfield T, Johnson MS. Citizen science for water quality monitoring: Data implications of citizen perspectives. *J Environ Manage*. 2017 9 15;200:456–67. 10.1016/j.jenvman.2017.05.083 28618317

[22] Selvam A, Kwok K, Chen Y, Cheung A, Leung KS, Wong JW. Influence of livestock activities on residue antibiotic levels of rivers in Hong Kong. *Environ Sci Pollut R*. 2017;24(10):9058–66. 10.1007/s11356-016-6338-5 26944426

[23] Zhou F, Liu Y, Guo HH. Application of Multivariate Statistical Methods to Water Quality Assessment of the Watercourses in Northwestern New Territories, Hong Kong. *Environ Monit Assess*. 2007 9;132(1–3):1–13. 10.1007/s10661-006-9497-x 17171256

